# Metabolomics and proteomics analyses revealed mechanistic insights on the antimicrobial activity of epigallocatechin gallate against *Streptococcus suis*

**DOI:** 10.3389/fcimb.2022.973282

**Published:** 2022-09-20

**Authors:** Ting Gao, Fei Ye, Yiqing Tan, Mingzheng Peng, Fangyan Yuan, Zewen Liu, Danna Zhou, Keli Yang, Wei Liu, Rui Guo, Tengfei Zhang, Lin Zheng, Rui Zhou, Yongxiang Tian

**Affiliations:** ^1^ Key Laboratory of Prevention and Control Agents for Animal Bacteriosis (Ministry of Agriculture and Rural Affairs), Hubei Provincial Key Laboratory of Animal Pathogenic Microbiology, Institute of Animal Husbandry and Veterinary, Hubei Academy of Agricultural Sciences, Wuhan, China; ^2^ Institute of Fruit and Tea, Hubei Academy of Agricultural Sciences, Wuhan, China; ^3^ State Key Laboratory of Agricultural Microbiology, College of Veterinary Medicine, Huazhong Agricultural University, Wuhan, China; ^4^ Pig disease prevention and control center, Cooperative Innovation Center of Sustainable Pig Production, Wuhan, China

**Keywords:** *Streptococcus suis*, tea polyphenols, EGCG, antibacterial, pathogenicity, Sly, metabolomics, proteomics

## Abstract

*Streptococcus suis* (*S. suis*) is a highly virulent zoonotic pathogen and causes severe economic losses to the swine industry worldwide. Public health security is also threatened by the rapidly growing antimicrobial resistance in *S. suis*. Therefore, there is an urgent need to develop new and safe antibacterial alternatives against *S. suis*. The green tea polyphenol epigallocatechin gallate (EGCG) with a number of potential health benefits is known for its antibacterial effect; however, the mechanism of its bactericidal action remains unclear. In the present, EGCG at minimal inhibitory concentration (MIC) showed significant inhibitory effects on *S. suis* growth, hemolytic activity, and biofilm formation, and caused damage to *S. suis* cells *in vitro*. EGCG also reduced *S. suis* pathogenicity in *Galleria mellonella* larvae *in vivo*. Metabolomics and proteomics analyses were performed to investigate the underlying mechanism of antibacterial activity of EGCG at MIC. Many differentially expressed proteins involved in DNA replication, synthesis of cell wall, and cell membrane, and virulence were down-regulated after the treatment of *S. suis* with EGCG. EGCG not only significantly reduced the hemolytic activity of *S. suis* but also down-regulated the expression of suilysin (Sly). The top three shared KEGG pathways between metabolomics and proteomics analysis were ABC transporters, glycolysis/gluconeogenesis, and aminoacyl-tRNA biosynthesis. Taken together, these data suggest that EGCG could be a potential phytochemical compound for treating *S. suis* infection.

## 1 Introduction

Tea is one of the most popular beverages because of its beneficial effects on human health (Cao et al., 2016). The major health-promoting ingredients of green tea are tea polyphenols and catechins, such as epigallocatechin-3-gallate (EGCG), epicatechin gallate (ECG), epigallocatechin (EGC), and epicatechin (EC) (Gao et al., 2022). Among these phytochemicals, EGCG is the most abundant tea polyphenols in green tea. Over the past two decades, EGCG has been studied for bactericidal properties, and it is particularly effective against Gram-positive bacteria, for instance, *Staphylococcus aureus, Solobacterium moorei, Bacillus subtilis, Streptococcus mutans* (Morin et al., 2015; Nakayama et al., 2015; Taylor et al., 2021; Zeferino et al., 2022). However, to date, no data exist regarding the effect of EGCG on cellular growth and viability of *Streptococcus suis*.

*S. suis* is an important zoonotic pathogen that infects both humans and swine and causes several infectious diseases such as meningitis, arthritis, pneumonia, and septicemia (Xia et al., 2019). Consequently, *S. suis* infection causes significant economic losses to the global swine industry and poses a threat to human health (Lun et al., 2007). To date, *S. suis* has been classified into 33 serotypes according to the different antigenicity of capsular polysaccharides; of these serotypes, *S. suis* serotype 2 (SS2) is identified as the most virulent and frequently isolated serotypes in swine and humans (Segura et al., 2017). Many virulence factors are responsible for the pathogenicity of *S. suis*, among which suilysin (Sly) is one of the important virulence factors (Xu et al., 2021). Sly is a hemolysin of *S. suis*, and it directly creates pores in target cellular membranes and aids bacterial colonization in the host (Lin et al., 2019). Sly contributes contributes extensively to the pathogenesis of *S. suis* infection and inflammatory response *in vitro* and *in vivo* (Tenenbaum et al., 2016). On the basis of this information, it could be postulated that a phytochemical compound which effectively inhibits Sly activity could reduce the pathogenicity of *S. suis* and relieve inflammatory response.

Over the last few decades, an increase in antimicrobial resistance has been reported in food-borne and other pathogens because of irrational use of antibiotics (Palmieri et al., 2011). This has been considered as a major public health issue, and worldwide emergence of multidrug-resistant bacteria has become a serious threat to human. Up to now, antimicrobial resistance *S. suis* becomes a major challenge in many endemic countries (Yongkiettrakul et al., 2019) such as Italy, Canada, the Netherlands, and China. Among 78 *S. suis* isolates recovered from diseased pigs in Italian farm during 2017 to 2019, 61.5% were resistant to two antibiotics and 29.5% were resistant to three antibiotics (Cucco et al., 2022). A total of 379 *S. suis* isolates obtained from Canada swine farms were tested for antimicrobial susceptibility, and most of these isolates were resistant to tetracycline, tiamulin, and spectinomycin (Arndt et al., 2019). Among 1,163 *S. suis* isolates recovered from the Netherlands during 2013 to 2015, 78.4% and 48.1% were resistant to tetracycline and clindamycin, respectively (van Hout et al., 2016). The drug sensitivity of 421 *S. suis* isolates from China was investigated and it was found that the antimicrobial resistance rates for tetracycline, macrolides, and sulfonamides were over 60% (Zhang et al., 2008). On the basis of these results, developing new and safe antibiotic alternatives against *S. suis* is urgently needed. EGCG extracted from edible medicinal plants is a potential drug that can be used as a novel strategy to control bacterial infection (Xiong et al., 2017) without promoting resistance. This strategy is compatible with the “One Health” approach that protects the health of both humans and animals and is also beneficial to sustainable development of ecological environment.

Thus far, the drug target of EGCG remains to be identified. The antibacterial effect of EGCG against *Pseudomonas aeruginosa* has been well studied, and previous studies have reported that EGCG might be a potential candidate for quorum sensing inhibition, biofilm formation reduction, and efflux pump control in *P. aeruginosa* (Kanagaratnam et al., 2017; Hao et al., 2021). In *Helicobacter pylori*, the bactericidal action of EGCG through another pathway was revealed, wherein EGCG interacted with histone-like DNA binding protein to efficiently kill the bacteria (Raj et al., 2021). In *Escherichia coli*, EGCG caused an increase in endogenous oxidative stress to inhibit bacterial growth; therefore, EGCG might be used as a prooxidant (Xiong et al., 2017). These reported functions indicate that EGCG possesses a broad spectrum of antibacterial activity.

In the present study, we investigated the antibacterial activity of EGCG against *S. suis in vitro* and *in vivo*. EGCG at minimum inhibitory concentration (MIC) not only showed significant inhibitory effects on *S. suis* growth, hemolytic activity, and biofilm formation and induced cellular damage, but it also protected *Galleria mellonella* larvae against *S. suis* infection (EGCG/larvae, 50mg/kg). Metabolomics and proteomics analyses were used to systematically investigate the underlying mechanism of antibacterial activity of EGCG at MIC. The results will provide scientific basis for the prevention and control of *S. suis* infection.

## 2 Materials and methods

### 2.1 EGCG extraction

EGCG was extracted from Chinese green tea processed by clonal tea leaves picked from *Camellia sinensis* “E’cha NO1” variety and analyzed by an e2695 high performance liquid chromatography (HPLC) (Waters, USA). EGCG (catalog number: 989-51-5; molecular weight; 458.37) used for calibration was purchased from Sigma-Aldrich Corporation (St Louis, USA). EGCG solution was prepared as follows: 131 mg of powder was dissolved in 1 mL of distilled water. The solution was then filtered through a 0.22 μm filter. Finally, culture medium was used to dilute the solution to appropriate concentration according to the experiments.

### 2.2 Bacterial strains and growth conditions

The virulent SS2 strain SC19 was isolated from a diseased pig in Sichuan, China, in 2005 (Li et al., 2009). For long-term preservation, the strain SC19 was maintained at -80°C after freeze-drying. *Escherichia coli* ATCC 25922 used as a reference strain in drug susceptibility tests was purchased from the China Center for Type Culture Collection. SC19 was grown in tryptic soy broth (TSB; BD, USA) or on tryptic soy agar (TSA; BD, USA) plates by adding 10% (v/v) newborn bovine serum (Sijiqing, Hangzhou, China) at 37°C. For drug susceptibility tests, the strain was were grown in Mueller-Hinton broth (MHB; BD, USA) or on MHB agar (MHA; BD, USA) at 37°C.

### 2.3 Time-killing curve of SC19 against EGCG

MIC and Minimum bactericidal concentration (MBC) were determined as described previously (Gu et al., 2020) in accordance with the guidelines of the Clinical and Laboratory Standards Institute (CLSI). The microdilution broth method was performed in 96-well plates. Briefly, the bacteria cells were grown in MHB to the mid-log phase, and the resulting cultures were dispensed into MHB supplemented with EGCG at concentrations ranging from 8192 to 16 µg/mL. The final concentration of the bacterial culture was 5×10^5^ CFU/ml.The MIC and MBC values were determined in three independent assays. The time-killing curve of EGCG against SC19 was determined as described previously (Morin et al., 2015). Growth curves of SC19 in EGCG at MIC, MBC were monitored by OD600 and CFU counts every 2 h for 18 h at 37°C.

### 2.4 Transmission electron microscope and scanning electron microscope (SEM) analysis of bacterial integrity

The effects of EGCG on bacterial integrity of SC19 were analyzed by transmission electron microscope (TEM) and scanning electron microscope (SEM) as described previously with minor modifications (Gao et al., 2020). The bacterial cells in the mid-log phase in TSB and EGCG at MIC were co-incubated at 37°C for 4 h, followed by fixation with 2.5% glutaraldehyde at 4°C overnight. The bacterial cells were then treated with 1% osmium tetroxide for 2 h at room temperature and dehydrated using a serial dilution of ethanol. For TEM, the dehydrated cells were embedded in epoxy resin and cell shape was analyzed by a HT7700 TEM (HITACHI, Japan). For SEM, the dehydrated cells were coated with a 10-nm-thick gold layer for 30 s and observed with a SU8100 SEM (HITACHI, Japan).

### 2.5 Effect of EGCG on hemolytic activity of SC19 culture supernatant

Hemolytic activity was tested as described previously with minor modifications (Wang et al., 2021). Briefly, the bacterial cells in the mid-log phase in TSB and EGCG at MIC and MBC were co-incubated at 37°C for 4 h, followed by collection of the culture supernatant by centrifugation at 12000 g for 2 min. The supernatant (100 µL) was then incubated with 2% sheep erythrocyte suspension (100µL) in PBS for 2 h at 37°C. Unlysed erythrocytes were removed, and the supernatant was collected by centrifugation at 1500 g for 15 min. Finally, the absorbance of the supernatant was measured at 543 nm by using a Victor Nivo multifunctional enzyme reader (PerkinElmer, USA). TSB was used as a negative control.

### 2.6 Biofilm inhibition assay

Biofilm inhibition assay was performed using regular 48-well micro-titer plates as described with some modifications (Hao et al., 2021). Briefly, the bacterial cells in the mid-log phase in TSB were cultured with EGCG at MIC and MBC for 48 h at 37°C. TSB was used as negative control. The cultures were then removed and the plates were washed three times with PBS. The formed biofilms were stained with 0.1% crystal violet for 30 min, and rinsed three times with double-distilled (dd) H^2^O and then dried in air. The biofilms bound to crystal violet were released in 200 µL of 95% alcohol. The absorbance was measured at 590 nm by Victor Nivo multifunctional enzyme reader (PerkinElmer, USA).

### 2.7 Protection of *Galleria mellonella* larvae by EGCG against SC19 infection

*G. mellonella* larvae were used as an infection model to evaluate the protection afforded by of EGCG (Loh et al., 2013). Each group comprised 10 larvae weighed 0.4 to 0.5g. Group 1 was administered 20 μL of PBS in the lower left proleg as the negative control. Group 2 was administered EGCG at the dose of 50 mg/kg in the lower left proleg (Gao et al., 2022; Li et al., 2022). Group 3 was administered EGCG at the dose of 50 mg/kg in the lower left proleg, and after 30 min, group 3 was challenged with a lethal dose of 2.0 × 10^6^ CFU of SC19 into the lower right proleg. Group 4 was administered a lethal dose of 2.0 × 10^6^ CFU of SC19 in the lower left proleg. The survival rate of insects in each group was monitored for 7 days.

### 2.8 Non-targeted metabolomics analysis

SC19 cells were cultured in TSB to the mid-log phase, and then co-incubated with EGCG at MIC at 37°C for 4 h. The resulting bacterial samples were centrifuged, then, the supernatant was discarded, and the bacterial pellet was mixed with 1 ml of cold methanol/acetonitrile (1:1, v/v) at -20°C for 1 h. The mixture was then treated with ultrasonication in ice and the resulting supernatant was dried under vacuum and re-dissolved in acetonitrile/water (1:1, v/v) solvent for analysis. Intracellular metabolomic determined were performed on an Agilent 1290 Infinity LC system (Agilent Technologies, Santa Clara, USA) combined with an Framingham AB SCIEX Triple TOF 6600 System (AB SCIEX, USA). The ESI source was under the following conditions: ion source gas1 (Gas1) as 60, ion source gas2 (Gas2) as 60, curtain gas (CUR) as 30, source temperature: 600°C, ionspray voltage floating (ISVF) ± 5500 V. In auto MS/MS acquisition mode, the instrument was set to acquire readings over the m/z range of 25-1000 Da, and the accumulation time for product ion scan was set at 0.05 s/spectrum (Chen et al., 2020).

The web-based system MetaboAnalyst was used for multivariate statistical analysis, including principal component analysis (PCA), partial-least squares discrimination analysis (PLS-DA) and orthogonal partial-least squares discrimination analysis (OPLS-DA). The significant different metabolites (DMs) were determined according to the VIP values and two-tailed Student’s t test of the raw data, and metabolites with VIP values > 1.0 and p values < 0.05 were considered significant.

### 2.9 Proteomics analysis

Samples preparation: The SC19 strain was cultured in TSB to the mid-log phase, and then co-incubated with EGCG at MIC at 37°C for 4 h. The resulting cultures were centrifuged and bacterial pellets were resuspended in SDT buffer (4% SDS, 100 mM Tris-HCl, 1 mM DTT, pH 7.6) and heated for 15 min at 100°C.

Protein extraction, digestion, TMT labeling, and LC-MS/MS analysis were performed as described previously (Gao et al., 2020). SC19 cells treated with EGCG were labeled with TMT tags 126, 127 and 128, while SC19 were labeled with tags 129, 130 and 131. In LC-MS/MS analysis, a reversed phase trap column-connected to a C18 reversed-phase analytical column was used for peptide mixture loading.

Proteomic data analysis was performed as described previously (Chen et al., 2020). Differentially expressed proteins (DEPs) were filtered out on the basis of the following criteria: p-value < 0.05, fold change > 1.2 (up regulation) or < 0.83 (down-regulation).

### 2.10 Combined metabolomics and proteomics analysis

All DEPs and DMs were queried and mapped to pathways based on the KEGG. R version 3.5.1 was used to combine KEGG annotation and enrichment result of metabolomics and proteomics. Venn diagrams and bar plots were drawn to combine the results of the two omics approaches.

### 2.11 Statistical analysis

Unless otherwise specified, the data were analyzed by two-tailed, unpaired t-test and all experiments were performed in triplicate at least thrice. All data were expressed as mean ± standard errors of the means (SEM), and p < 0.05 was considered the threshold for significance. Significant difference in survival between the different groups was analyzed by the log rank test. GraphPad Prism 7 was used to perform statistical analysis.

## 3 Results

### 3.1 Quantitative determination of EGCG and antimicrobial activity against SC19

**Figure 1:**
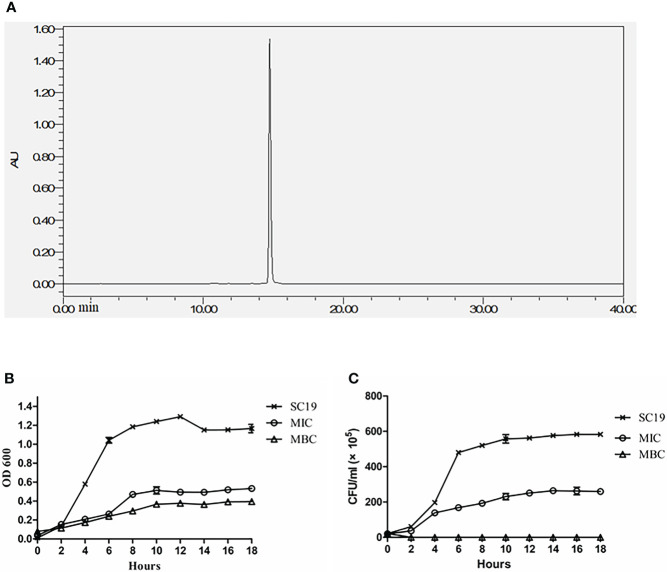
EGCG extract quantification and effect of EGCG on SC19 growth **(A)** The EGCG extract was quantitatively determined by HPLC, The purity of EGCG reached 99.99%. **(B)** Kinetics of the killing effect of EGCG on SC19. The concentrations of EGCG ranged from MIC to MBC. Bacterial viability were monitored by CFU counts at the indicated times. **(C)** OD 600 of SC19 in the absence of EGCG and in the presence of EGCG at MIC and MBC.

EGCG extract was quantitatively identified by HPLC. The purity of EGCG was 99.99% (**Figure 1A**). As reported in **Table 1**, the MIC and MBC levels of EGCG on SC19 were 512 and 1024 μg/mL, respectively. In contrast, the MIC and MBC levels of EGCG against the reference strain *E. coli* ATCC25922 were 4096 and 8192 μg/mL, respectively.

**Table 1:** MIC and MBC values of EGCG against SC19 and ATCC25922.

Strains	Compoud	MIC (µg/ml)	MBC (µg/ml)
ATCC25922	EGCG	4096	8192
SC19	EGCG	512	1024

### 3.2 EGCG effectively killed virulent SC19 strain *in vitro*

Time-killing curves were constructed evaluate the bactericidal effect of ECGC on SC19. As shown in **Figures 1B, C** EGCG effectively killed *S. suis* cells in a dose-dependent manner, and the bacterial growth was inhibited at the MIC of 512 μg/mL of EGCG as compared to untreated cells. After 2 h of co-incubation, the bacteria cells were completely killed at the MIC of 1024 μg/mL of EGCG.

### 3.3 Destructive effect of EGCG on bacterial internal and external structure

**Figure 2:**
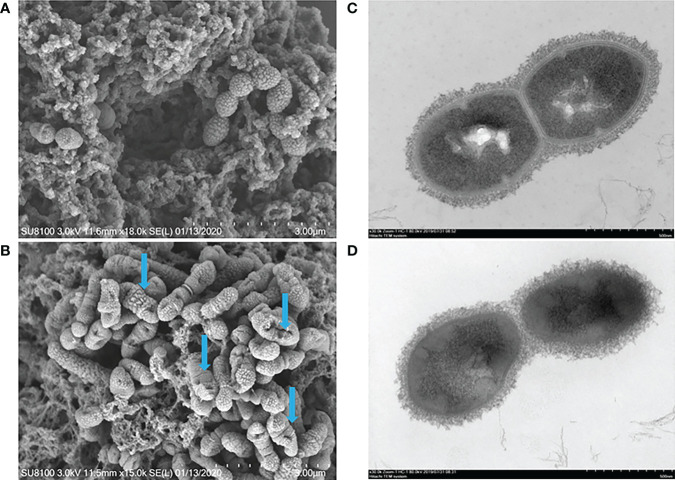
SEM and TEM analysis of SC19. **(A)** Untreated bacteria control analysis *via* SEM, the bar at the bottom right means 3 μm. **(B)** Bacteria treated with EGCG (512 μg/ml) analysis *via* SEM, the bar at the bottom right means 3 μm. **(C)** Untreated bacteria control analysis *via* TEM, the bar at the bottom right means 500 nm. **(D)** Bacteria treated with EGCG (512 μg/ml) analysis *via* TEM, the bar at the bottom right means 500 nm. The blue arrow indicated cellular damage caused by EGCG.

The effect of EGCG on the morphology of SC19 was directly visualized by scanning electron microscopy and transmission electron microscopy. Scanning electron microscopy observations revealed that *S. suis* cells were severely shrunken after treatment at the final concentration of 512 μg/mL EGCG, while untreated cells were coccoid or ovoid in shape and normally occurred as single cells, in pairs, or in short chains (**Figures 2A, B**). Moreover, most cells lost their shape owing to the leakage of cytoplasmic content. Transmission electron microscopy showed that EGCG treated cells exhibited heterogeneous electron density in the cytoplasm, indistinct cell wall, collapsed cell membrane and plasmolysis (**Figures 2C, D**).

### 3.4 EGCG decreased hemolytic activity and biofilm formation of SC19 and protected *Gmellonella larvae* against SC19 infection

**Figure 3:**
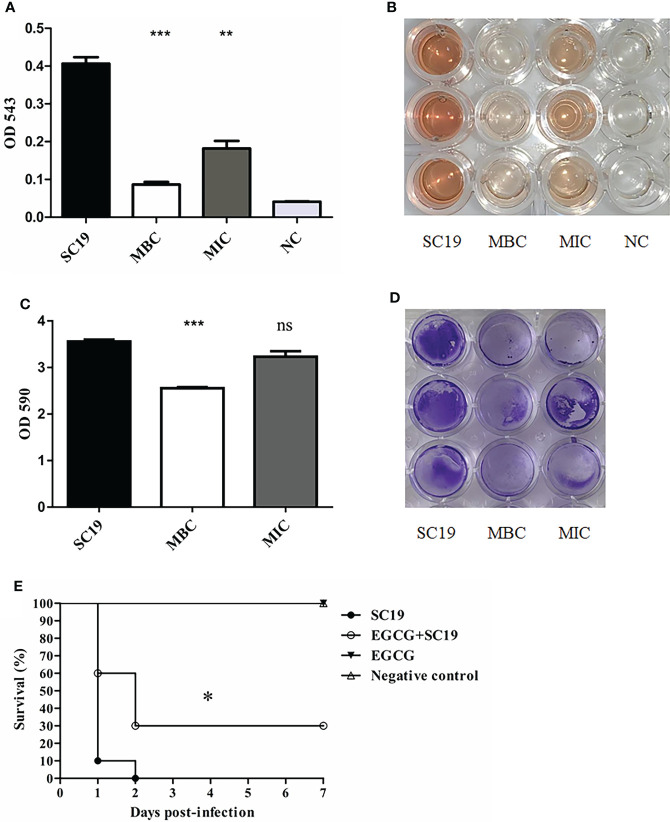
Hemolytic activity, biofilm formation and infection experiment and of SC19 affected by EGCG. **(A)** Hemolytic activity analysis of SC19 affected by EGCG. Absorption was measured at 543 nm to determine Sly production. TSB was used as negative control. **(B)** Microplate showing difference of hemolytic activity between EGCG treated SC19 and untreated SC19 **(C)** Biofilm formation analysis of SC19 affected by EGCG. Absorption was measured at 590 nm to determine biofilm production. TSB was used as negative control. **(D)** Microplate showing difference of biofilm formation between EGCG treated SC19 and untreated SC19. **(E)** Survival curves for *Galleria mellonella* larvae in experiment infection. Significant difference in survival between different groups was analyzed by Log Rank test. The height of the bars indicates the mean values for the relative expression data ± SEM (ns, p > 0.05; *p < 0.05; **p < 0.01; ***p < 0.001).

The hemolytic assay showed that EGCG markedly reduced hemolytic activity of SC19 at MIC (p<0.01). When the concentration of EGCG increased to MBC, hemolytic activity of SC19 was four times less than that for the untreated group (p <0.001), thus indicating that EGCG decreased hemolytic activity of SC19 culture supernatants in a dose-dependent manner (**Figures 3A, B**).

The results for biofilm formation revealed that EGCG significantly reduced the formation of SC19 biofilms at MBC (p α0.001), but it had no impact at MIC (p<0.05) (**Figures 3C, D**).

Previous studies have shown that tea polyphenols could confer protection against pathogenic bacteria *in vivo* (Guo et al., 2018; Ma et al., 2021). Therefore, the larvae infection model was used to analyze the bactericidal effect of EGCG *in vivo*. As expected, larvae in group 1 and 2 were normal and showed 100% survival. Larvae in group 4 died within 48 h of the infection and exhibited complete melanization. However, larvae in group 3 administered with 50 mg/kg EGCG before infection showed mild clinical symptom, and 30% of the infected larvae survived after 48 h (**Figure 3E**).

### 3.5 Effects of EGCG on metabolic profiling of SC19

**Figure 4:**
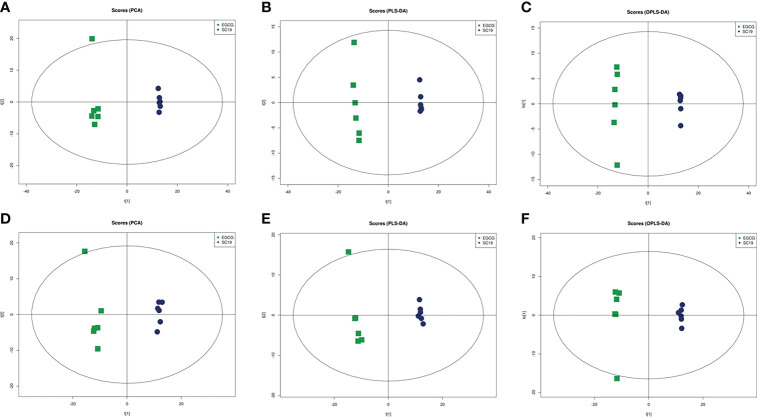
Differentiation of the metabolic profiles of the SC19 vs EGCG treated SC19 using multivariate analysis. PCA analysis of metabolites under positive **(A)** and negative **(D)** ion modes. PLS-DA analysis of metabolites under positive **(B)** and negative **(E)** ion modes. OPLS-DA analysis of metabolites under positive **(C)** and negative **(F)** ion modes. Spots in blue show samples from the SC19 group, spots in green indicate samples from the EGCG treated SC19 group, there are six replicates per group.

**Figure 5:**
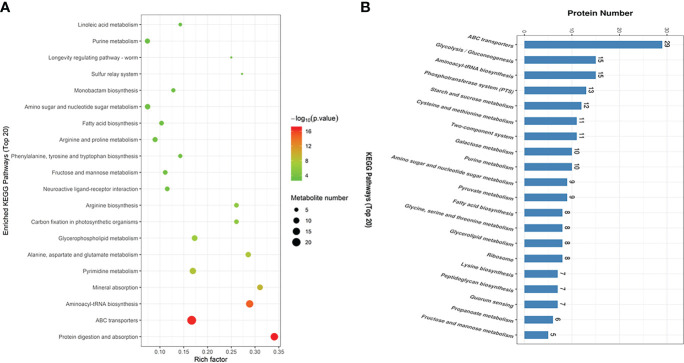
The enriched KEGG pathways of the DMs and DEPs in SC19 compared to EGCG treated SC19. **(A)** Enrichment analysis of the differential metabolites. Rich factor represents the ratio of the number of DMs to total metabolites in each pathway. **(B)** Enrichment analysis of the differently expressed proteins. The top 20 KEGG pathways were shown on the graph.

A total of 11592 and 11607 ion peaks under positive and negative modes were collected, respectively. The PCA scores plot (positive: R^2^X = 0.699; negative: R^2^X = 0.663), PLS-DA scores plot (positive: R^2^X = 0.665, R^2^Y = 0.999, Q^2^ = 0.989; negative: R^2^X = 0.639, R^2^Y = 0.998, Q^2^ = 0.984), and OPLS-DA scores plot (positive: R^2^X = 0.665, R^2^Y = 0.999, Q^2^ = 0.992; negative: R^2^X = 0.639, R^2^Y = 0.998, Q^2^ = 0.986) showed a clear distinction between the two groups in both positive and negative modes (**Figure 4**). A total of 121 DMs with significant differences were identified in the EGCG -treated group, among which 75 were up-regulated (fold change > 1.5) and 46 were down-regulated (fold change < 0.67). EGCG greatly affected SC19 metabolites associated with amino acids, nucleotides, amino sugars, vitamins, and so on. KEGG enrichment analysis was performed to further analyze the relationship of interaction between EGCG and SC19. The top 20 perturbed enriched pathways were protein digestion and absorption, ABC transporters, aminoacyl-tRNA biosynthesis, mineral absorption pyrimidine metabolism, alanine, aspartate and glutamate metabolism, glycerophospholipid metabolism, carbon fixation in photosynthetic organisms, arginine biosynthesis, fatty acid biosynthesis, and so on (**Figure 5A**).

### 3.6 Effects of EGCG on the proteome of SC19

To further investigate the mechanism underlying the changes in metabolites after EGCG treatment, a proteomics analysis with TMT labeling was used to investigate the changes in the proteome of SC19. The MS proteomics data have been deposited in the ProteomeXchange Consortium through the PRIDE (Perez-Riverol et al., 2019) partner repository under the dataset identifier PXD026047. A total of 2,026 proteins were detected and quantified according to the database, among which 500 were DEPs (almost 24.7% of expressed proteins) in EGCG treated SC19 cells as compared to that in untreated SC19 cells, with 277 up regulated DEPs and 223 down regulated DEPs (p < 0.05, fold change > 1.2). Of these DEPs, many were involved in cell growth and division, cell wall and membrane composition, drug resistance, environmental adaption, and hemolytic activity (**Table 2**).

**Table 2:** List of the identified DEPs between SC19 vs EGCG treated SC19.

Protein name	Functions		Ratio (EGCG/SC19)		Uniquepeptides	Sequence coverage (%)
**DNA replication associated**						
Smc	Chromosome partition protein		3.154588		1	38.91
PolC	DNA polymerase III		1.830461		2	27.68
DnaK	Chaperone protein		0.497069		1	79.9
DnaG	DNA primase		1.991800		1	26.51
RpoB	DNA-directed RNA polymerase subunit beta		1.897211		1	57.56
DnaB	Replication initiation/membrane attachment protein		0.81552		11	30.55
A6M16_07230	Helicase		0.765107		6	50.39
Smc	Chromosome partition protein		1.717061		1	39.42
Fic	Cell filamentation protein		2.418688		1	23.51
FtsA	Cell division protein FtsA		2.256282		1	60.66
**Cell wall associated**						
MurE	UDP-N-acetylmuramoyl-L-alanyl-D-glutamate–L-lysine ligase		2.154161		1	30.56
MurAB	UDP-N-acetylglucosamine 1-carboxyvinyltransferase		2.035757		1	51.79
Pbp2A	Penicillin-binding protein 2A		1.21914		24	40.98
MurB	Site-specific tyrosine UDP-N-acetylenolpyruvoylglucosamine reductase		0.743356		9	33.44
	Segregation and condensation protein B		0.740106		6	36.48
PbpD	Membrane carboxypeptidase (Penicillin-binding protein)		0.709299		2	37.92
	Cell wall biosynthesis glycosyltransferase		0.394956		1	19.53
ERS132461_00734	LPXTG cell wall surface protein		1.582084		1	2.72
ERS132461_02224	Cell wall surface anchor family protein		1.240508		1	1.11
**Cell membrane associated**						
ERS132414_00279	Membrane protein	2.123565		1		3.33
ID09_11505	Membrane protein	1.737502		1		6.17
ERS132394_00175	Membrane protein	1.470201		2		16.95
ERS132531_00304	Membrane protein	1.379608		2		6.48
ID09_01605	Membrane protein	0.734679		2		9.94
ID09_07730	Membrane protein	0.729151		6		43.52
ID09_02880	Membrane protein	0.686472		1		2.5
ID09_03715	Membrane protein	0.604128		2		10.49
LI88_12640	Membrane protein	0.585303		2		7.22
A6M16_05425	Multidrug ABC transporter ATP-binding protein	0.656875		1		29.03
yheH_2	Multidrug ABC transporter ATPase and permease	0.654701		1		30.73
yheI_2	Multidrug ABC transporter ATPase/permease	0.802254		18		37.22
tetM	Tetracycline resistance	0.746043		12		18.78
PyrP	Xanthine/uracil permease	0.530648		1		4.3
GuaB	Inosine-5’-monophosphate dehydrogenase	0.742405		1		80.12
**Virulence associated**						
NeuC	UDP-N-acetylglucosamine 2-epimerase	0.825171		17		55.17
Sly	Thiol-activated cytolysin	0.616023		8		15.58
Cps2B	Chain length determinant protein	0.831134		4		52.84
Cps2E	Galactosyl transferase	0.769827		12		23.75
Cps2G	Glycosyltransferase	0.792496		16		50.65
Cps2J	Glycosyltransferase	0.760726		12		34.88
Cps2K	Glycosyltransferases involved in cell wall biogenesis	1.240508		13		47.83
Cps2R	Acetyltransferase	0.72227		5		28.37
**Environmental adaption associated**						
Ndk	Nucleoside diphosphate kinase	0.535681		1		44.53
HPr	Phosphocarrier protein	0.824924		1		59.26
CspA	Cold-shock protein	0.552037		5		58.21
RelA/SpoT	GTP pyrophosphokinase	1.468818		11		47.53

According to gene ontology (GO) analysis using Blast2GO, 500 DEPs were classified into three categories: molecular function, biological process, and cellular component. The main molecular functions included acetyltransferase activity, transferase activity, transferring acyl groups other than amino-acyl groups, N-acetyltransferase activity, N-acyltransferase activity, and passive transmembrane transporter activity. The main biological process contributed to the regulation of cellular protein metabolic process, regulation of translation, posttranscriptional regulation of gene expression, regulation of cellular amide metabolic process, and regulation of protein metabolic process. Regarding cellular component, most DEPs were related to membrane, extracellular region, cell, protein containing complex, membrane part, and cell part (**Figure S1**). GO enrichment analysis were performed using Fisher’s exact test (p < 0.05)with the whole quantified protein annotations as the background dataset. Seventeen GO terms listed in **Table 3** were significantly perturbed by EGCG treatment.

**Table 3:** GO enrichment on ontologies for biological process and molecular function of SC19 treated by EGCG.

GO ID	Term	Category	Test protein	Reference protein	*p*-value		Richfactor	
GO:0016407	acetyltransferase activity	molecular function	13	27	0.006584		0.48	
GO:0016747	transferase activity, transferring acyl groups other than amino-acyl groups	molecular function	18	43	0.009233		0.42	
GO:0008080	N-acetyltransferase activity	molecular function	10	20	0.012246		0.50	
GO:0016410	N-acyltransferase activity	molecular function	10	20	0.012246		0.50	
GO:0022803	passive transmembrane transporter activity	molecular function	5	8	0.025597		0.63	
GO:0015267	channel activity	molecular function	5	8	0.025597		0.63	
GO:0016746	transferase activity, transferring acyl groups	molecular function	18	48	0.031494		0.38	
GO:0016798	hydrolase activity, acting on glycosyl bonds	molecular function	12	30	0.045082		0.40	
GO:0032268	regulation of cellular protein metabolic process	biological process	4	5	0.014773		0.80	
GO:0006417	regulation of translation	biological process	4	5	0.014773		0.80	
GO:0010608	posttranscriptional regulation of gene expression	biological process	4	5	0.014773		0.80	
GO:0034248	regulation of cellular amide metabolic process	biological process	4	5	0.014773		0.80	
GO:0051246	regulation of protein metabolic process	biological process	4	5	0.014773		0.80	
GO:0006865	amino acid transport	biological process	3	4	0.048825		0.75	
GO:0015711	organic anion transport	biological process	3	4	0.048825		0.75	
GO:0046942	carboxylic acid transport	biological process	3	4	0.048825		0.75	
GO:0015849	organic acid transport	biological process	3	4	0.048825		0.75	

To further investigate the effect of DEPs in the cell physiological process and discover internal relationships between the DEPs, KEGG enrichment analysis was performed. The top 20 KEGG pathways are listed in **Figure 5B**, including ABC transporters, glycolysis/gluconeogenesis, aminoacyl-tRNA biosynthesis, phosphotransferase system (PTS), starch and sucrose metabolism, cysteine and methionine metabolism, two-component system, galactose metabolism, purine metabolism, amino sugar and nucleotide sugar metabolism, pyruvate metabolism, and fatty acid biosynthesis.

### 3.7 Combined metabolomics and proteomics analyse of EGCG-treated SC19 cells

**Figure 6:**
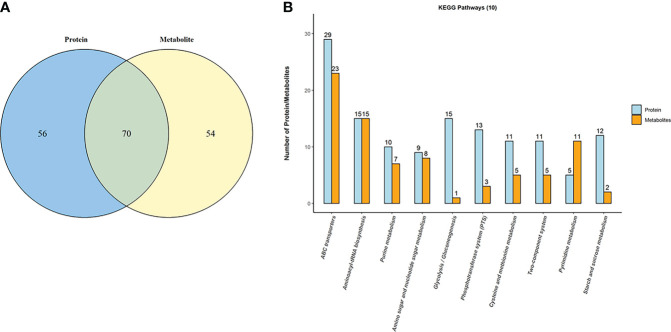
Combination analysis of metabolomics and proteomics for EGCG treated SC19. **(A)** Venn diagram depicting the overlap between metabolites and proteins. **(B)** The top 10 shared KEGG pathways were shown on the graph.

To reveal the underlying regulatory mechanism through which EGCG exerted its bactericidal activity against SC19, we performed a combined metabolomics and proteomics analysis. Seventy 70 shared KEGG pathways were noted between metabolomics and proteomics analyse as shown in Venn diagram (**Figure 6A**). As shown in **Figure 6B**, the top 10 KEGG pathways included ABC transporters, glycolysis/gluconeogenesis, aminoacyl-tRNA biosynthesis, PTS, starch and sucrose metabolism, cysteine and methionine metabolism, two-component system, galactose metabolism, purine metabolism, amino sugar and nucleotide sugar metabolism, pyruvate metabolism, fatty acid biosynthesis, and so on.

## 4 Discussion

Several studies have determined the bactericidal activity of green tea polyphenols against *Pseudomonas aeruginosa, Listeria monocytogenes, Staphylococcus aureus, Escherichia coli, and Streptococcus mutants* and have revealed the antibacterial mechanism of EGCG, which involves inhibiting virulence factors such as toxins and extracellular matrix molecules, damaging the cell membrane, and so on. All these activities make EGCG to be a promising antibiotic agent for treating bacterial infections (Steinmann et al., 2013; Stenvang et al., 2016). However, the drug target of EGCG is yet to be identified, and the effect of EGCG on bacterial metabolomics and proteomics has not yet been systematically studied.

In the present study, EGCG was purified from E’cha No.1 variety of green tea and quantified by HPLC. To the best of our knowledge, the present study is the first report on EGCG functions as a bactericidal drug against SC19. The MIC and MBC values of EGCG against SC19 were lower than those of EGCG against *E. coli* ATCC 25922, thus indicating that Gram positive SC19 was more sensitive to EGCG than Gram negative *E. coli* ATCC 25922. The cell membrane of Gram-positive bacteria is positively charged, whereas EGCG is negatively charged; thus, they can bind to each other (Reygaert, 2014; Reygaert, 2018). In contrast, Gram-negative bacteria are more resistant to EGCG because of the presence of lipopolysaccharide in the outer membrane (Xiong et al., 2017).

EGCG also reduced biofilm formation and pathogenicity of SC19 and induced cellular damage; this finding is consistent with the report of a previous study. In the present study, cellular ultrastructural changes were accompanied by deep cell surface shrinkage (blue arrows indicate the damaged and lysed SC19 cells) and intracellular content leakage after treatment with EGCG at MIC. It is likely that at a higher concentration, the bactericidal effects of EGCG led to bacterial cell lysis. Broken cells and cell debris were visible in TEM and SEM observations (Zhu et al., 2021). As described in the Introduction section, the antibacterial mechanism of EGCG was reviewed, and the present study provided more information on the antibacterial activity of EGCG. EGCG also decreased the hemolytic activity of SC19. Sly, which induces hemolytic activity, is an essential virulence factor for *S. suis* as it creates pores in the target cells membranes (Wang et al., 2021). *S. suis* strains with high levels of Sly production are more likely to cause high mortality in infected mice than non-virulent strains, thus indicating that the pathogenicity of *S. suis* can be attenuated by lowering the production of Sly (Takeuchi et al., 2014). In the present study, it was unclear whether decreased hemolytic activity was due to the inhibition or due to the reduced expression of Sly. A decrease in the viable count of bacteria or a decrease in the expression of the Sly protein could decrease hemolytic activity. To elucidate the reason for decreased hemolytic activity, proteomics analysis was performed, and Sly was found to be down-regulated in SC19 after exposure to EGCG. We believe that the effect of EGCG on decreased hemolytic activity of SC19 is because of the down-regulated Sly or due to the effect of EGCG on growth inhibition. In the present study, EGCG showed efficient biofilm inhibition of *S. suis*; this may be due to down-regulation of DEPs (5/6) involved in the synthesis of capsular polysaccharides (CPS). CPS contribute to the adherence of pneumococci to host cells (Wang et al., 2017), and, bacterial adhesion is the first essential step for biofilm formation. Like most pathogens, the ability of *S. suis* to form biofilms also plays an essential role in its virulence and drug resistance (Wang et al., 2018). Gilbert reported that biofilm-associated cells were 10–1,000 times more resistant to antimicrobial agents than planktonic cells due to a decreased penetration of antibiotics (Gilbert et al., 1997). In the proteomics analysis, six DEPs associated with drug resistance were down-regulated, including multidrug ABC transporter ATP-binding protein (A6M16_05425), multidrug ABC transporter ATPase and permease (YheH_2), multidrug ABC transporter ATPase/permease (YheI_2), tetracycline resistance (TetM), Xanthine/uracil permease (PyrP), and Inosine-5’-monophosphate dehydrogenase (GuaB). ABC transporters are membrane proteins that participate in the influx or efflux of various molecules (Megías-Vericat et al., 2022). In addition to these six membrane proteins, there are also nine membrane proteins and seven cell wall proteins were differentially expressed, most of which were down-regulated. Moreover, through TEM and SEM observations, we noted indistinct cell wall and collapsed cell membrane after exposure to EGCG. On the basis of these results, we speculated that EGCG affects the functions of the cell membrane and cell wall.

We then performed the first study of combining metabolomics and proteomics to reveal the mechanisms underlying the antibacterial activity of EGCG against SC19. Through comparison of DEPs with DMs, 70 KEGG pathways were found to be shared in the two omics approaches, as, shown in **Figure 6A**. In particular, ABC transporters were the top one perturbed KEGG pathway after treatment with EGCG. Efflux pump is a type of ABC transporters, and overexpression of efflux pumps in P. aeruginosa is one of the most important mechanisms involved in intrinsic and acquired resistance to antibiotics (Goli et al., 2016). A previous study indicated that the antibacterial activity of EGCG was associated with the expression of efflux pump (Kanagaratnam et al., 2017). The results also suggested that EGCG may act as an efflux pump inhibitor in *P. aeruginosa*. In the present study, six multidrug ABC transporter associated DEPs were down-regulated. On the basis of these findings, we speculated that the weak expression of these DEPs enhanced the susceptibility of SC19 cells to EGCG. This may also suggest EGCG synergistically interacted with the enhanced susceptibility of SC19 to antibiotics. Aminoacyl-tRNA biosynthesis needs aminoacyl-tRNA synthetases, which have evolutionarily conserved mechanisms for protein synthesis (Coker et al., 2022) and are detrimental to cellular viability. Recent studies suggest that in some instances such changes facilitate adaption to stress conditions. Hence, the perturbation in the aminoacyl-tRNA biosynthesis pathway suggests that EGCG impacts environmental adaptability and damages the bacterial cell, thus providing some clues to elucidate the effect of EGCG on SC19. In addition, four DEPs (Ndk, HPr, CspA, RelA/SpoT) associated with environmental adaption were identified through proteomics analysis, among which RelA/SpoT was up-regulated when SC19 cells were exposed to EGCG. RelA/SpoT enzymes control bacterial physiology through the synthesis and degradation of the nucleotide alarmone (p)ppGpp (Kurata et al., 2021). When, bacterial cells encounter environmental stresses, such as cold, nitrogen limitation, salinity stress, and astaxanthin exposure, RelA/SpoT expression is significantly up-regulated to adapt to the new environment (Jin et al., 2022). Therefore, on the basis of these observations, we speculate that EGCG may regulate the expression of ABC transporters to allow EGCG influx into SC19 cells and affect the normal metabolic activity of these channels. On the other hand, because of the influx of EGCG, SC19 cells were under stress conditions and up-regulated the expression of RelA/SpoT to synthesize the alarmones pGpp, ppGpp, and pppGpp as a stringent response for adapting to antibiotic tolerance, biofilm formation, production of secondary metabolites, or virulence (Horvatek et al., 2020).

## 5 Conclusions

In conclusion, the results of the present study showed that EGCG at MIC exhibited significant inhibitory effects on *S. suis* growth, hemolytic activity, and biofilm formation and caused damage to bacterial cells in vitro. EGCG also protected *G. mellonella* larvae against *S. suis* infection *in vivo*. Metabolomics and proteomics analyses were combined to determine the underlying mechanism of the antibacterial activity of EGCG at MIC. Many proteins associated with DNA replication, cell wall, cell membrane and virulence were down-regulated after EGCG treatment. In particular, EGCG at MIC not only significantly reduced hemolytic activity of S. suis but also down-regulated the expression of Sly. The top three perturbed KEGG pathways were ABC transporters, glycolysis/gluconeogenesis, aminoacyl-tRNA biosynthesis. Taken together, these data suggest that based on its antibacterial and antihemolytic activity, EGCG can be a potential phytochemical for treating *S. suis* infection.

## Data availability statement

The datasets presented in this study can be found in online repositories. The names of the repository/repositories and accession number(s) can be found in the article/**Supplementary Material**.

## Author contributions

TG and FY conceived and designed this project and experiments. YQT, MP, ZL, and WL performed the experiments. DZ, KY, FYY, LZ and RG analyzed the data. TG, FY, TZ and YQT contributed to the development of the figures and tables. TG, RZ and YXT wrote the manuscript. All authors reviewed the manuscript. All authors contributed to the article and approved the submitted version.

## Funding

This research was funded by the National Key Research and Development Program of China (2021YFD1800401), the Natural Science Foundation of China (NSFC; Grant No. 31802189), the Hubei Province Natural Science Foundation for Innovative Research Groups (2021CFA019), China Agriculture Research System of MOF and MARA (CARS-19), the Technical Innovation Project of Hubei Province (2021ABA005), the Science and Technology Project of Hubei Province (2020BBA038), and the Hubei Province Innovation Center of Agricultural Sciences and Technology (2019-620-000-001-017, 2019-620-000-001-24).

## Acknowledgments

We would like to thank Shanghai Applied Protein Technology Co. Ltd. for providing technical support.

## Conflict of interest

The authors declare that the research was conducted in the absence of any commercial or financial relationships that could be construed as a potential conflict of interest.

## Publisher’s note

All claims expressed in this article are solely those of the authors and do not necessarily represent those of their affiliated organizations, or those of the publisher, the editors and the reviewers. Any product that may be evaluated in this article, or claim that may be made by its manufacturer, is not guaranteed or endorsed by the publisher.
