# Supplementary figures and images for: Metabolomics and proteomics analyses revealed mechanistic insights on the antimicrobial activity of epigallocatechin gallate against Streptococcus suis
Source: Front Cell Infect Microbiol. 2022 Sep 20;12:973282. doi: 10.3389/fcimb.2022.973282 (PMC9531131; doi:10.3389/fcimb.2022.973282)

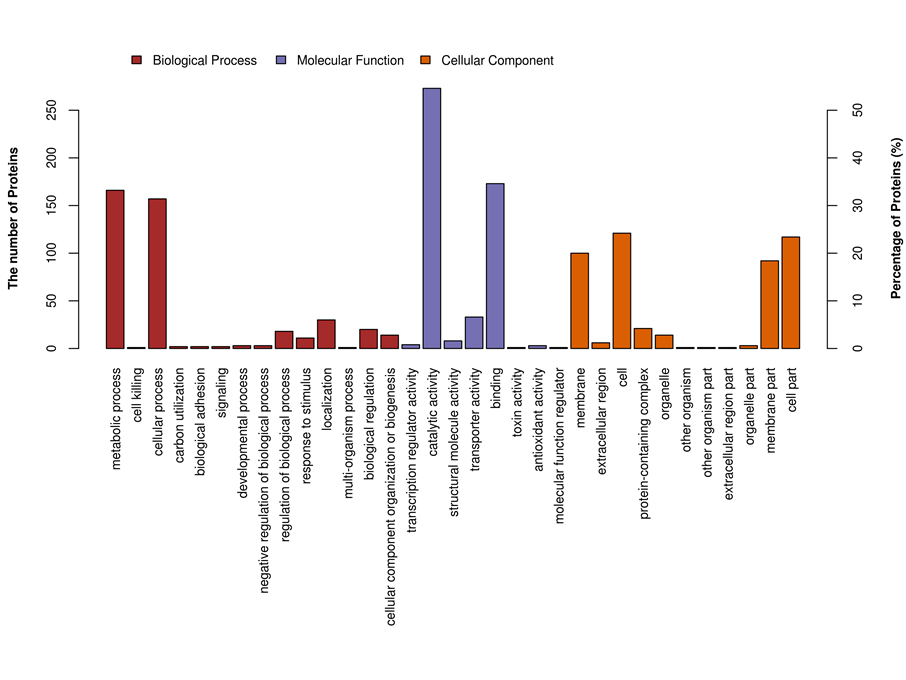

Supplement: Supplementary file 1 [file Image_1.tif]
